# ClC Channels and Transporters: Structure, Physiological Functions, and Implications in Human Chloride Channelopathies

**DOI:** 10.3389/fphar.2017.00151

**Published:** 2017-03-23

**Authors:** Diogo R. Poroca, Ryan M. Pelis, Valérie M. Chappe

**Affiliations:** ^1^Department of Physiology and Biophysics, Dalhousie University, HalifaxNS, Canada; ^2^Department of Pharmacology, Dalhousie University, HalifaxNS, Canada

**Keywords:** ClC channels, myotonia congenita, leukodystrophy, salt loss, deafness, Dent’s disease, osteopetrosis, channelopathy

## Abstract

The discovery of ClC proteins at the beginning of the 1990s was important for the development of the Cl^-^ transport research field. ClCs form a large family of proteins that mediate voltage-dependent transport of Cl^-^ ions across cell membranes. They are expressed in both plasma and intracellular membranes of cells from almost all living organisms. ClC proteins form transmembrane dimers, in which each monomer displays independent ion conductance. Eukaryotic members also possess a large cytoplasmic domain containing two CBS domains, which are involved in transport modulation. ClC proteins function as either Cl^-^ channels or Cl^-^/H^+^ exchangers, although all ClC proteins share the same basic architecture. ClC channels have two gating mechanisms: a relatively well-studied fast gating mechanism, and a slow gating mechanism, which is poorly defined. ClCs are involved in a wide range of physiological processes, including regulation of resting membrane potential in skeletal muscle, facilitation of transepithelial Cl^-^ reabsorption in kidneys, and control of pH and Cl^-^ concentration in intracellular compartments through coupled Cl^-^/H^+^ exchange mechanisms. Several inherited diseases result from C1C gene mutations, including myotonia congenita, Bartter’s syndrome (types 3 and 4), Dent’s disease, osteopetrosis, retinal degeneration, and lysosomal storage diseases. This review summarizes general features, known or suspected, of ClC structure, gating and physiological functions. We also discuss biophysical properties of mammalian ClCs that are directly involved in the pathophysiology of several human inherited disorders, or that induce interesting phenotypes in animal models.

## Introduction

Ion transporters typically use the electrochemical gradient of one substrate (or another source of energy such as ATP) to transport another substrate in a well-defined stoichiometry and direction. This is a relatively slow process limited by the number of ions/substrate that can bind to the transporter in a given transport cycle, as well as by the need for conformational changes to deliver the transported substrate to the opposing side of the membrane. Conversely, ion channels passively move ions down electrochemical gradients at a high-rate flux through a pore with a defined selectivity.

Ion channels commonly exist in four states: closed, open, inactive, and desensitized, with each state having a different ion conductance. “Gating” is the term used to describe changes between the different states. Various factors, including voltage, ligand binding, second messengers, volume, and temperature modulate ion channel gating (Kew and Davies, 2010). Because of their important role in cell function, mutations in ion channel genes that cause impaired channel function are associated with a variety of human diseases, generally known as channelopathies. Channelopathies affect the nervous, cardiovascular, respiratory, endocrine, urinary, and immune systems.

Many years of intense research have focused on how changes in ion channels’ biophysical properties can induce drastic physiological changes at the cellular and tissular levels, subsequently causing severe and even lethal human diseases. The ultimate goal of this research is the development of specific targeted pharmacotherapies to treat channelopathies. Perhaps the most well-known example of such a therapy is the treatment of cystic fibrosis patients with corrector and potentiator drugs that specifically target the Cystic Fibrosis Transmembrane conductance Regulator (CFTR) chloride channel to alleviate mutations affecting its trafficking, folding, and function. Although this extraordinary translational development is still in its infancy, with less than 4 years of clinical use, it has required over two decades of research to reach the necessary level of understanding of the biophysical and functional parameters of the CFTR chloride channel.

Prior to cloning of the first chloride channels (ClC-0 and CFTR), chloride channels were of little interest to scientists, as Cl^-^ was considered to be in electrochemical equilibrium across cell membranes. When studying action potentials, cation channels (Na^+^, K^+^, and Ca^2+^) were considered the major players; Cl^-^ flux was seen as a mere nuisance. Chloride movement across membranes can change both the concentration of the substrate, Cl^-^, and the electrical charge between the compartments. As an electronegative ion, chloride plays an important role in regulating the excitability of neurons and muscles through changes in the membrane potential. In epithelia, the Cl^-^ concentration gradient drives the direction of ion movement through ion transporters, which helps maintain intra- and extra-cellular osmotic homeostasis. The cloning of the CFTR chloride channel (Riordan et al., 1989) and the *Torpedo* electric organ voltage-gated chloride channel ClC-0 (Jentsch et al., 1990) were important breakthroughs in chloride channel research, paving the way for subsequent high impact publications on chloride transport. ClC-0 was not characteristic of any other chloride transporter previously described, and thus became the first member of the new ClC chloride channel family. Nine mammalian ClC proteins have been identified since the discovery of ClC-0. Four of these ClCs are expressed in the plasma membrane and operate as channels (**Table 1**), and the other five are Cl^-^/H^+^ exchangers localized to intracellular membranes (**Table 2**). The ClC family of Cl^-^ transporters is the focus of this review.

**Table 1:** Mammalian ClC chloride channels.

	Isoform	Tissue	Function	Human disease	Knock-out mice
Cl^-^ channels (cell surface)	ClC-1	Skeletal muscles	Recover resting membrane potential	Myotonia congenita	Myotonia congenita (Steinmeyer et al., 1991a)
	ClC-2/ (GlialCAM)	Brain; kidney; liver; heart; pancreas; skeletal muscles; lungs and GI tract	Transepithelial transport	Leukodystrophy, azoospermia	Retinal and testes degeneration; leukodystrophy (Bösl et al., 2001)
	ClC-Ka/Barttin	Inner ear; Kidney	Transepithelial transport	Loss of Barttin or both ClC-Ks: Bartter IV (renal salt loss and deafness	Diabetes insipidus (Matsumura et al., 1999)
	ClC-Kb/Barttin			Loss of ClC-Kb: Bartter III (renal salt loss)	

*The table illustrates tissue expression, function and pathologies related to dysfunction or absence of ClC channels from plasma membranes. Both ClC-K a and b isoforms require Barttin as an obligatory β-subunit for trafficking, stability and function. GlialCAM is a non-essential β-subunit of ClC-2 and, when associated, change ClC-2 localization and properties in glial cells.*

**Table 2:** Mammalian ClC chloride exchangers.

	Isoform	Tissue	Function	Human disease	Knock-out mice
Cl^-^/H^+^ exchangers Intracellular (Endo/lysosomes)	ClC-3	Broad	Ion homeostasis of intracellular vesicles		Retinal and brain degeneration (Stobrawa et al., 2001; Dickerson et al., 2002; Yoshikawa et al., 2002)
	ClC-4	Skeletal muscles; brain and heart	Ion homeostasis of endosomes?	Intellectual disabilities?	
	ClC-5	Kidney; intestine	Ion homeostasis of early endosomes	Dent’s disease	Impaired renal endocytosis (Piwon et al., 2000; Wang et al., 2000)
	ClC-6	neurons	Ion homeostasis of late endosomes		Lysosomal storage disease (Poët et al., 2006)
	ClC7/ Ostm1	Brain; kidney; liver and bone	Acidification of resorption lacuna in osteoclasts; ion homeostasis of lysosomes	Osteopetrosis, retinal degeneration, lysosomal storage disease	Osteopetrosis; retinal degeneration; lysosomal storage disease (Kornak et al., 2001)

*The table illustrates tissue expression, function and pathologies related to defect or absence of the respective ClC exchangers from vesicles of the endosomal/lysosomal pathway. ClC-7 needs Ostm1 as an essential β-subunit to be stable and functional at lysosomal membranes.*

## The ClC Family

The discovery of *Torpedo* ClC-0 channel by Jentsch et al. (1990) garnered the attention of the scientific community toward the ClC protein family. ClC proteins occur in all phyla, with nine members present in mammals (ClC-1 to ClC-7, ClC-Ka, and ClC-Kb). Three of the ClC proteins contain a β-subunit (ClC-Ka, ClC-Kb, and ClC-7), which is essential for proper transport function, and another member (ClC-2) contains a non-essential β-subunit that changes its gating properties (Jentsch, 2015).

Although Jentsch et al. (1990) cloned the first ClC member, the Miller group discovered most of the surprising and unique properties of this family prior to this. Observations of single channel current recordings from *Torpedo electroplax* Cl^-^ channels demonstrated an unusual gating behavior, with bursts containing two open conductance levels spaced by long periods of channel closing (Miller, 1982; Hanke and Miller, 1983). Since one conductance level was the double of the other, they assumed that the channel functions as a dimer, with each subunit having its own independent ion pathway (protopore). Gating of one or both subunits’ protopores explained the two distinct conductance levels observed; meanwhile, the single closed state suggested that despite the two protopores functioning independently, some as-yet-unknown mechanism closed them simultaneously (Richard and Miller, 1990). In this now well-established double-barrel model there is a fast gate (opening and closing events within a burst) occurring on a time scale of milliseconds and a slow (also called common) gate, in which both protopores are closed on a time scale of seconds, reflecting the single closed state observed in single channel analysis. All channel properties identified by Miller’s group were afterward attributed to the ClC family of chloride channels.

In ClCs—unlike cation channels, whose gating is regulated by voltage sensors controlled by the membrane potential—the permeant ion (Cl^-^) is itself responsible for the voltage-dependent gating, and protons influence the gating (Richard and Miller, 1990; Pusch et al., 1995; Bezanilla, 2008). That is, intra- and extra-cellular changes in Cl^-^ concentration and pH modulate ClC channel function. In general, ClC channels have an anion selectivity sequence of Cl^-^ > Br^-^ > I^-^ and are largely impermeable to cations (Jentsch et al., 1995; Fahlke et al., 1997a,b; Rychkov et al., 1998).

In another surprising discovery, researchers have determined that while all ClC proteins share the same basic structure, some function as chloride-proton exchangers with a 2Cl^-^/1H^+^ stoichiometry, instead of classical chloride channels (Accardi and Miller, 2004; Picollo and Pusch, 2005; Scheel et al., 2005). In mammals, five ClC proteins function as Cl^-^/H^+^ exchangers (ClC-3 to ClC-7) and are generally localized to intracellular membranes, while the other four (ClC-1, ClC-2, ClC-Ka, and ClC-Kb) function as bona fide chloride channels, strictly localized to the plasma membrane.

Malfunctions in chloride conductance or Cl^-^/H^+^ translocation are causes of genetically inherited diseases (Puljak and Kilic, 2006; Planells-Cases and Jentsch, 2009; Kim, 2014; Stölting et al., 2014b; Jentsch, 2015).

### ClC Protein Structure

In 2002, high-resolution crystal structures of two bacterial ClC exchangers were resolved (EcClC from *E. coli* and StClC from *S. typhimurium*). Exhibiting a complex topology, each ClC subunit has 18 α-helices that are variable in length and remarkably tilted. Most of the α-helices fail to traverse the membrane and display an internal anti-parallel repeat architecture. This intriguing arrangement of helices makes it possible for residues from distant parts of the protein to come together at the center of the subunit, forming the ion selectivity filter for Cl^-^ conductance (Dutzler et al., 2002, 2003).

In Dutzler’s StClC structure, each ClC subunit has three highly conserved Cl^-^ binding sites, which feature a partial positive charge formed by amino acid residues located in the N-terminal portion of specific α-helices (D, F, N, and R). In the crystal structure, Cl^-^ could be found at three specific sites made up by these amino acids: (1) an internal site (S_int_) in contact with the intracellular environment, (2) a central site (S_cen_) buried in the membrane bilayer, and (3) an external site (S_ext_) in contact with the extracellular solution. In this structure, S_int_ and S_cen_ are occupied by Cl^-^ ions, whereas S_ext_ is occupied by the negatively charged side-chain of a conserved glutamate (E148; helix F) named Glu_ext_. In S_cen_, Cl^-^ ions are coordinated mainly by residues S107 (helix D) and Y445 (helix R), also called Ser_cen_ and Tyr_cen_, respectively. A Cl^-^ ion occurs in S_ext_ only following mutation or protonation of E148, which renders ClC gating proton-dependency. Importantly, mutation of this glutamate residue (E148Q, which mimics protonation of the carboxylate side chain) abolishes voltage and chloride-dependent gating in ClC channels and uncouples Cl^-^/H^+^ exchange, turning the proteins into passive chloride conductors (Dutzler et al., 2003; Accardi and Miller, 2004). E148 has been termed the ‘gating glutamate,’ given its essential role in ClC protein function.

Some researchers have proposed that Cl^-^ and E148 compete for S_ext_, and that Cl^-^ conductance (during the pore opening) occurs only when the side-chain of E148 is displaced from S_ext_ by extracellular Cl^-^ (Chen, 2003). Presumably, this is the reason that ClC gating is dependent on extracellular Cl^-^ concentration. While the ‘gating glutamate’ in the S_ext_ is suggested to be the molecular determinant of protopore gating (Dutzler et al., 2003), S107 in the S_cen_ is thought to contribute to Cl^-^ selectivity, as mutation of this residue to proline changes anion selectivity to NO_3_^-^ (Zifarelli and Pusch, 2009). S_int_ is located close to where the intracellular solution bathes the selectivity filter, and residues in helix D coordinate Cl^-^ ions in this position (Dutzler et al., 2003).

For ClC exchangers to function, a proton pathway is also required, although there is currently no consensus on how protons cross the transport pathway. A glutamate residue (E203), located at the intracellular interface (named Glu_int_) is suggested to be the proton acceptor coupling H^+^ and Cl^-^ transport, as mutation of this residue abolishes proton transport (Accardi et al., 2005). Glu_ext_ is conserved in both channels and exchangers and is involved in both Cl^-^ and H^+^ conductance, whereas Glu_int_ is only conserved in exchangers and participates only in H^+^ transport (Accardi and Miller, 2004; Accardi et al., 2005). Concurrent mutation of the intracellular and extracellular glutamates leads to a loss of proton transport, although Cl^-^ transport is still active. Glu_int_ localizes away from the Cl^-^ selectivity filter, in a region closer to the subunit’s interface. Although experimental data is lacking, Glu_int_ and Glu_ext_ appear to cooperate to facilitate proton transport. In the proposed mechanism, Glu_int_ accepts a H^+^ from one side of the membrane and transfers it to Glu_ext_, which then completes the translocation process (Accardi et al., 2005). However, it is not clear how protons would traverse the gap between Glu_int_ and Glu_ext_, and because of Glu_int_ localization, the pathways for Cl^-^ and H^+^ would diverge in the intracellular side converging only in the extracellular side, at Glu_ext_.

The first relatively high-resolution structure of a mammalian ClC channel (a bovine ClC-K) was solved by cryo-electron microscopy (Park et al., 2016). Bovine ClC-K (henceforth, bClC-K) shares 84% sequence similarity with human ClC-K channels and is only functional when co-expressed with the β-subunit barttin. bClC-K contains a valine residue (V166) substituted for Glu_ext_, which causes the channel to have a linear current–voltage relationship (Park et al., 2016). Based on sequence homology the structure of bClC-K is predicted to be similar to other ClC family members (Dutzler et al., 2002, 2003; Feng et al., 2010). However, the high-resolution structure reported by Park et al. (2016) suggests some marked differences. bClC-K contains two extracellular loops, one connecting helices K and M and the other connecting helices I and J. Both loops are located at the extracellular entrance of the chloride pathway with the latter loop in close proximity to the Cl^-^ selectivity filter. There is also a cytosolic loop connecting helices C and D that displays a unique conformation from ClC transporters. In bClC-K the loop contains Ser_cen_ (S121 in ClC-K), which faces the cytosolic side, whereas in ClC transporters Ser_cen_ is found facing S_cen_ interacting with a Cl^-^ ion together with Tyr_cen_ (Park et al., 2016). Based on these structural differences between bClC-K and ClC transporters the authors propose a hypothesis for the different mechanisms underlying ClC channel and transporter conductance. They suggest that in transporters Ser_cen_ and Tyr_cen_ form a kinect barrier (a constriction) in the middle of the Cl^-^ pathway preventing Cl^-^ leak uncoupled to H^+^ during a transport cycle. In ClC channels they suggest that the unique positioning of Ser_cen_ relieves the kinect barrier allowing higher Cl^-^ conductance (Park et al., 2016).

The most well studied plant ClC protein is the anion/proton exchanger AtClC-a, from *Arabidopsis thaliana*. AtClC-a promotes the exchange of NO_3_ rather than Cl^-^ due to a substitution of Ser_cen_ to proline (Zifarelli and Pusch, 2009). AtClC-a shows outwardly rectifying currents and a 2NO_3_/1H^+^ stoichiometry when expressed in isolated vacuoles, similar to animals and prokaryotes ClC exchangers (De Angeli et al., 2006). Glu_ext_ and Glu_int_ are conserved in AtClC-a and inactivation of those residues produces similar effect as in the intracellular ClCs 4 and 5 (Bergsdorf et al., 2009).

#### Cytoplasmic Domains

All eukaryotic ClCs (and some prokaryotic ClCs) have a large cytoplasmic domain involved in modulating the trafficking and function of ClC proteins (Estévez et al., 2004; Hebeisen et al., 2004). Mutations in the cytoplasmic domains cause severe defects in slow gating, and are also associated with human genetic diseases (Fong et al., 1998; Estévez et al., 2004; Puljak and Kilic, 2006; Planells-Cases and Jentsch, 2009; Kim, 2014; Stölting et al., 2014b). The crystal structures of cytoplasmic domains from ClC-0, ClC-Ka, and ClC-5 have been resolved; cytoplasmic domains of each subunit contain two CBS domains that interact with one another via an extensive interface. The CBS domains also interact with the transmembrane part of the same subunit, and with CBS domains of the other subunit. Additionally, the cytoplasmic domains display a dimeric organization resembling the transmembranal architecture (Meyer and Dutzler, 2006; Markovic and Dutzler, 2007; Meyer et al., 2007; Feng et al., 2010; Park et al., 2016).

The cytoplasmic domains connect with the α-helix R, which contains Tyr_cen_ that participates directly in Cl^-^ coordination during transport. As mutations in the cytoplasmic domains are involved in genetic diseases, several studies have addressed the influence of alterations in the cytoplasmic domains in channel gating behavior. A point mutation downstream of the second CBS domain (A885P) in ClC-1 results in a dramatic reduction in channel open probability at voltages near the optimal membrane potential for ClC-1 to function (Beck et al., 1996). Two truncated ClC-1 mutants (R875X and K894X), the first removing the whole region downstream of CBS2 and the second mimicking a naturally occurring mutation in myotonic patients, display changes in anion binding affinity, resulting in changes in the voltage dependence for both fast and slow gates (Hebeisen and Fahlke, 2005). He et al. (2006) analyzed two splice variants of *Caenorhabditis elegans* ClC channel, CLH3a and CLH3b, which display marked gating differences. CHL3a has a N-terminal splice insertion that when deleted do not alter gating properties. CHL3b has two splice insertions at the cytoplasmic domains, one between the two CBS domains and the second distal to CBS2. Deletion of either the insertion distal to CBS2 or the last 11 amino acids of CBS1 gives rise to channels with gating properties similar to CHL3a (He et al., 2006). Those studies demonstrate that alterations at the cytoplasmic domains modify the conformation of the pore affecting channel gating.

Cytoplasmic domains of some ClC proteins interact with adenosine nucleotides. In ClC-1, binding of intracellular ATP inhibits the channel by stabilizing it in its closed state (Bennetts et al., 2005). ATP binding has the opposite effect in ClC-5 exchangers, activating the transporter. Binding of ATP to ClC-2 slows down the rate of activation and deactivation, but does not affect the maximal open probability of the channel (Stölting et al., 2013). Nucleotides bind at the interface between the two CBS domains, as revealed by the crystal structure of ClC-5. The nucleotide binding site has no catalytic properties, and to it AMP, ADP, and ATP bind with equal affinity (Meyer et al., 2007). There are no apparent nucleotide binding sites in the cytoplasmic domains of ClC-0 or ClC-Ka (Meyer and Dutzler, 2006; Markovic and Dutzler, 2007). Interestingly, the cytoplasmic domains of the plant ClC AtClC-a also interacts with adenosine nucleotides. At this exchanger, ATP reduces transport activity by a maximum of 60%. Unlike ClC-5, only ATP produces this effect, with AMP working only as a competitor limiting ATP inhibition when present in solution (De Angeli et al., 2009).

The antiparallel dimerization observed with the CBS domains of ClC proteins following ATP binding is a feature also seen in the CFTR chloride channel, in which ATP binds at two conserved motifs at the interface of two intracellular nucleotide binding domains. These domains dimerize in a head-to-tail conformation leading to channel gating and chloride movement following conformational changes. By analogy, one may speculate that adenosine nucleotide binding to the CBS domains could cause protein rearrangements that affect channel behavior in some ClCs.

#### Common Gating

In contrast to the well-studied fast gating mechanism of ClC proteins, the molecular mechanism of the slow (common) gate is still obscure. Evidence suggests that extensive conformational rearrangements in the protein could contribute to the slow gate (Pusch et al., 1997; Duffield et al., 2005; Bykova et al., 2006; Ma et al., 2011). Two facts support the idea that conformational changes promoted by the cytoplasmic domains may lead to the movement of critical transmembrane helices and play an important role in the common gating mechanism. First, point mutations at helices localized at the dimer interface cause changes in common gating (Duffield et al., 2003). Second, the crystal structure of the eukaryotic ClC transporter show relevant connections between the CBS domains and helices H and I, localized at the dimer interface (Feng et al., 2010).

Another interesting hypothesis suggests that ClC channels likely behave as ‘broken’ exchangers in which proton transport is involved in the common gating, suggesting that the conformational changes of channels’ common gating and coupled Cl^-^/H^+^ transport have an evolutionary linkage (Lísal and Maduke, 2008). Since gating of ClC-0 channels is not in a thermodynamic equilibrium (Richard and Miller, 1990), the authors demonstrated that proton transport is involved in ClC-0 gating and is, in fact, the source of energy that keeps ClC channels in this asymmetric gating state (Lísal and Maduke, 2008). Further support for this position comes from a study in which a small but reproducible H^+^ transport demonstrated in ClC-1 channels was no longer identified in ClC-0 channels carrying the C212S mutation that abolish common gating (Picollo and Pusch, 2005).

There is evidence for a critical role for the Glu_ext_ residue in this mechanism (Dutzler et al., 2003; Cederholm et al., 2010), which would make this residue an essential part of both gating processes in ClC channels and also in the Cl^-^/H^+^ ion translocation in ClC exchangers. Feng et al. (2010) proposed a hypothesis for the mechanism of coupled Cl^-^/H^+^ transport, in which Glu_ext_ cycles between S_ext_, S_cen_, and the extracellular environment. While occupying S_cen_, Glu_ext_ interacts with Tyr_cen_ and accepts a proton from the intracellular H^+^ pathway. Then, following a conformational change after protonation, it would deliver the H^+^ to the extracellular solution. Presupposing that common gating and Cl^-^/H^+^ translocation are evolutionarily linked, and using the Cl^-^/H^+^ transport mechanism described above as a model, Bennetts and Parker (2013) suggested that Glu_ext_ and Tyr_cen_ play an important role for ClC-0 and ClC-1 common gating as they do for Cl^-^/H^+^ translocation. Additionally, they proposed that conformational changes for closure of the common gating involve helices G, F, H, I, and the CBS2 domain of the adjacent subunit, resulting in an arrangement that places Glu_ext_ (helix F) in position for hydrogen bonding with Tyr_cen_ (helix R), locking the channel closed. In this model, helix G would function as the coordinator between protopore and subunit interface, integrating both subunits for the common gating.

In the same work, the authors reported the involvement of Tyr_cen_ in Zn^+2^ inhibition and NAD^+^ modulation of the common gate (Bennetts and Parker, 2013). This research sheds some light on the molecular determinants of the common gating of ClC channels, but much remains unclear. The pathway for the H^+^ transport—proposed to be involved in the common gating—is not yet defined, as the suggested intracellular coordinator (Glu_in_) is changed by a valine residue in ClC channels. Also, in the eukaryotic CmClC Cl^-^/H^+^ exchanger, Glu_in_ is replaced by a threonine residue that either perform this transport or this exchanger would use an alternative H^+^ pathway (Feng et al., 2010). A neighboring conserved Glu residue (E291 in ClC-1), however, was proposed as a substitute to execute this function (Lísal and Maduke, 2009). Mutation to a protonable aspartate (E291D) shifted voltage dependence to more positive values but preserved the pH dependence, whereas mutation to a neutral glutamine (E291Q) remarkably reduced voltage and pH dependence, suggesting the participation of this residue in the H^+^ transport (Lísal and Maduke, 2009). This assumption, however, cannot be confirmed based solely on mutagenesis experiments. The exact molecular rearrangement necessary for the common gating is another puzzle, with many parts still missing.

**FIGURE 1:**
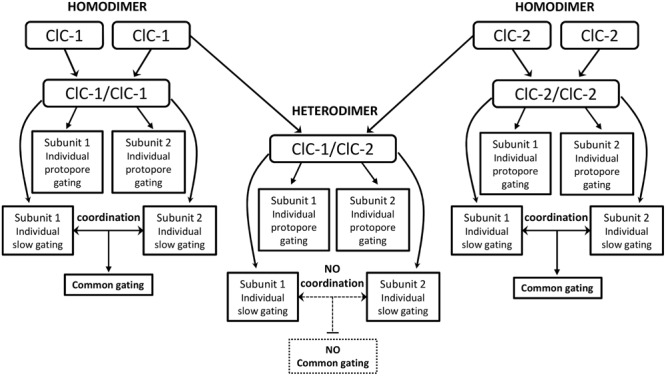
**Flowchart of the proposed new gating behavior of ClC-1/ClC-2 heterodimers (Stölting et al., 2014a).** Homodimers present individual fast gating for each subunit and a single common gating generated by the coordination of each subunit’s slow gating. In the heterodimer assembly (center), the individual protopore gating is maintained whereas coordination of each subunit’s slow gating is missing. In those channels each subunit displays individual slow gating (with distinct time and voltage dependence), therefore, the common gating is not observed.

One recent study analyzing ClC-1/ClC-2 heterodimeric channels revealed channels with original gating properties. The common gating was abolished, with each subunit displaying individual slow gates as well as independent fast gates (Stölting et al., 2014a). These findings suggest that conformational changes underlying common gating mechanisms may originate within each protopore gate, and that fast and slow gating may in fact be linked mechanisms (Bennetts and Parker, 2013; Stölting et al., 2014a). Homodimeric channels are able to coordinate both slow gates, resulting in a single common gating, whereas heterodimeric channels lack this coordination and display individual slow gating for each subunit (**Figure 1**).

### Importance of ClC Channels and Exchangers in Cell Homeostasis

ClC proteins are important for a number of physiological processes. In skeletal muscle, sodium and potassium channels provide the influx and efflux of cations necessary for propagation of the action potential, and the Cl^-^ current generated by ClC-1 is critical for proper re-polarization of the muscle fiber (Steinmeyer et al., 1991a; Stölting et al., 2014b). Impairment of ClC-1 function leads to myotonia, a condition characterized by delays in muscle relaxation after a contraction (Planells-Cases and Jentsch, 2009; Imbrici et al., 2015). ClC-2 in enterocytes and ClC-Kb in the thick ascending limb of Henle’s loop, working in concert with Na^+^-K^+^-ATPase, are necessary for Na^+^ and Cl^-^ transport from the lumen to the interstitium (Uchida et al., 1995; Estévez et al., 2001; Catalán et al., 2004). In the inner ear, K^+^ accumulation in the endolymph (critical for inner hair cell sensory transduction) mediated by KCNQ1/KCNE1 K^+^ channels is only possible because ClC-K channels recycle the Cl^-^ accumulated into the cell (by the action of Na^+^-K^+^-Cl^-^ co-transporter) back to the interstitial fluid (Rickheit et al., 2008).

In vesicular membranes of the endosomal/lysosomal pathway, Cl^-^/H^+^ exchange mediated by ClCs is required for vesicular acidification, which is necessary for endocytosis, vesicle sorting and lysosomal digestion (Piwon et al., 2000; Hara-Chikuma et al., 2005a; Kasper et al., 2005; Lange et al., 2006; Weinert et al., 2010). In all tissues and subcellular compartments described above, and many others, ion channel and transporter activity combines to maintain homeostasis. Dysfunction in even one component of the system can lead to drastic ion imbalances that may culminate in local or systemic diseases. In the case of ClC proteins, impaired function of ClC-1, ClC-2, ClC-K, ClC-5, and ClC-7 may result in myotonia congenita, azoospermia/leukodystrophy, Bartter syndromes types 3 and 4, Dent’s disease, and osteopetrosis/retinal degeneration/lysosomal storage disease, respectively.

## Mammalian ClCs and Human Disorders

### ClC-1: A Skeletal Muscle Chloride Channel

ClC-1 was the first mammalian ClC channel identified using homology cloning from the *Torpedo* ClC-0 channel. ClC-1 is expressed almost exclusively in skeletal muscle (Steinmeyer et al., 1991b), and has the same double-barreled conformation reported for ClC-0, although with a considerably smaller conductance. Activation of the fast and slow gating of ClC-1 requires depolarization that is dependent on the Cl^-^ and H^+^ concentration (Fahlke et al., 1996; Saviane et al., 1999). Adenosine nucleotides’ inhibition of CLC-1 is regulated by oxidation and reduction. Thus, ATP inhibits only reduced ClC-1 channels by shifting the voltage-dependence of common gating to more positive potentials; this inhibition disappears upon oxidation of ClC-1 (Zhang et al., 2008). Nucleotides bind in a putative site formed by residues from both CBS domains, and this inhibition is enhanced by low intracellular pH (Bennetts et al., 2005, 2012). This may be the mechanism by which the muscle fiber regulates channel function depending on the metabolic state. PKC and Zn^+2^ were also found to modulate ClC-1 function. Blocking by Zn^+2^ is closely related to the slow gating process. Mutation C277S locks the slow gate opened and abolishes the Zn^+2^ blocker effect, whereas mutation V321A reduces slow gating opening and facilitates Zn^+2^ blocking, suggesting that the effect of this ion is dependent on the state of the slow gating (Duffield et al., 2005). Several serine residues were identified in the C-terminal portion of ClC-1 that may mediate PKC modulation of the channel function (Hsiao et al., 2010). PKC activators inhibit the channel, whereas PKC inhibitors increase the current, suggesting that PKC phosphorylation of the C-terminal portion is important for ClC-1 function (Brinkmeier and Jockusch, 1987; Tricarico et al., 1991; Camerino et al., 2014).

#### ClC-1 and Myotonia Congenita

**FIGURE 2:**
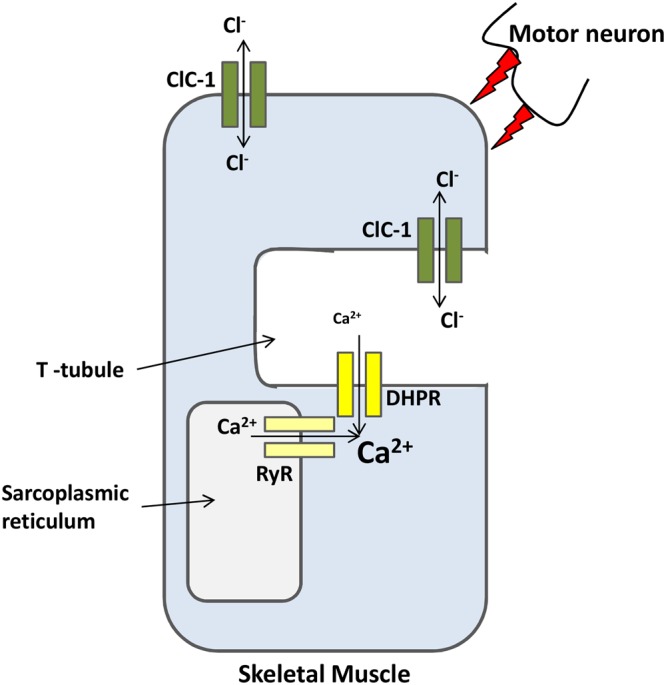
**ClC-1 is a major ion channel involved in the membrane resting potential of skeletal muscles.** Action potentials, from motor neurons, causes the opening of L-type calcium channels (DHPR) that in turn open intracellular channels (RyR). Calcium release from both channels increases sarcoplasmic reticulum [Ca^2+^] necessary for muscle contraction. After contraction, K^+^ efflux repolarizes the membrane. ClC-1 chloride conductance prevents K^+^ accumulation at the T-tubules from propagating along the sarcolemma and trigger undesirable autonomous depolarizations.

Skeletal muscle has a uniquely high resting Cl^-^ conductance that is more than four times greater than the K^+^ conductance (Bretag, 1987). ClC-1 is the predominant mediator of the Cl^-^ conductance in skeletal muscle. The first insight into the physiological role of ClC-1 came from studies using myotonic goats (Lipicky and Bryant, 1966) and myotonic *adr* mice (Steinmeyer et al., 1991a). Skeletal muscle fibers from these animals failed to repolarize following repeated action potentials, resulting in the so-called ‘myotonic after-discharge’ condition, characterized by muscle stiffness (Adrian and Bryant, 1974). After an action potential, Na^+^ channels close and K^+^ channels open to allow the ion efflux necessary for repolarization. In the T-tubules this K^+^ accumulation (increasing of [K^+^]_ext_) may generate small depolarizations even after inputs from the nervous system have ceased. ClC-1 mediates Cl^-^ conductance that prevents the K^+^-mediated depolarization from propagating along the sarcolemma (**Figure 2**). In myotonic fibers, the lack of ClC-1 conductance leads to autonomous fiber action potentials that keep the muscle active, delaying relaxation (Steinmeyer et al., 1991a; Stölting et al., 2014b). Mutations in the ClC-1 gene were found in families with myotonia congenita. These mutations lead to partial or complete loss of function of ClC-1, affecting channel function in different ways depending on the mutation. A group of mutations cause a reverted voltage dependency, i.e., D136G (Fahlke et al., 1995), G499R (Zhang et al., 2000), C277Y (Weinberger et al., 2012), G523D (Ha et al., 2014). These mutations cause the channels to activate upon hyperpolarization rather than deactivate like wild-type ClC-1, rendering channels with dramatically reduced or abolished currents at physiological chloride gradients. Mutation G230E (Fahlke et al., 1997a) and the aforementioned C277Y alters the ion selectivity of the channel pore. The A531V has normal gating properties but has reduced expression at the plasma membrane due to an increased degradation rate (Lee et al., 2013). It was later shown by Chen et al. (2015) that a ubiquitin ligase complex (CUL4A/B-DDB1-CRBN) ubiquinates the A531V mutant leading to its subsequent degradation. To date, more than 130 mutations have been identified in the gene encoding ClC-1, and heterologous expression of mutated channels has played a valuable role in helping scientists to understand channel structure and function and disease pathogenesis (Matthews et al., 2010; Imbrici et al., 2015).

Myotonia congenita is the most common skeletal muscle hereditary channelopathy in humans, characterized by an atypical delay in muscle relaxation after voluntary contractions, called muscle stiffness. The myotonic stiffness is worse after rest, and improves after repetitive movements, referred as the warm-up phenomenon (Thomsen, 1876; Bryant, 1973). In humans, myotonia can be inherited as a dominant (Thomsen disease) or a recessive (Becker disease) trait, with more severe symptoms found in the latter form (Saviane et al., 1999). In the dominant form, mutant subunits exert a dominant negative effect on wild-type subunits; that is, the mutant impairs (at variable levels) the function of the wild-type subunit.

Using the crystal structure of cmClC (an eukaryotic ClC exchanger) (Feng et al., 2010) as a model for human ClC-1 allowed the identification of residues found mutated in myotonic patients in the dimer interface and in the ion conduction protopore (Skálová et al., 2013). Moreover, mutations causing the dominant-negative effect were located in or proximal to the dimer interface region. Meanwhile, mutations affecting the channel protopore do not exert the dominant-negative effect (Skálová et al., 2013). As the slow gating involves subunit interactions at the dimer interface, mutations affecting this area in only one subunit prevent the coordination necessary for the common gating and explain the impairment of the adjacent wild-type subunit. In the recessive form, both subunits are affected and ClC-1 currents may be abolished completely, leading to the more severe symptoms reported (Saviane et al., 1999; Imbrici et al., 2015).

To date, there is no specific treatment for patients with myotonia congenita. To surpass ClC-1 defect, the ideal drug should specifically enhance its Cl^-^ currents; unfortunately, this objective seems to be far from completion (Imbrici et al., 2015). One early study showed that the R-isomer of CPP, a clofibric acid derivative, was able to increase Cl^-^ conductance in voltage clamp recordings of muscle fibers (De Luca et al., 1992). This activity was not recognized in heterologously expressed channels, suggesting that the drug does not interact directly with ClC-1 and probably uses a muscle-specific component to exert its effect (Pusch et al., 2000). Acetazolamide (a carbonic anhydrase inhibitor) was also reported to be able to shift the voltage-dependence of ClC-1 channel opening to more negative voltages, possibly through changes in intracellular pH, consequently enhancing Cl^-^ conductance. However, this potentially anti-myotonic effect was not effective in some mutant channels (Eguchi et al., 2006; Desaphy et al., 2013).

### ClC-2: A Widely Expressed ClC Channel

The discovery of ClC-2 came soon after ClC-1. ClC-2 is approximately 50% identical to ClC-1, and is expressed in the plasma membrane of cells from a variety of tissues, including the brain, kidney, pancreas, skeletal muscles, heart, lungs, gastro-intestinal tract, and liver (Thiemann et al., 1992). ClC-2 opens in a very short time course upon hyperpolarization. Its voltage-dependent gating is modulated by the concentration of Cl^-^ and H^+^. Increase in the intracellular concentration of Cl^-^ shifts the voltage-dependence to a more positive voltage, activating the channel. ClC-2 is also activated by mild decreases in extracellular pH, although a further decrease in pH reduces current (Niemeyer et al., 2004).

ClC-2 can bind to the accessory molecule GlialCAM, an adhesion molecule, in several glial cell types. This interaction is not required for the channel to function, but rather modifies the channel gating properties (Jeworutzki et al., 2012). GlialCAM also binds to Mlc1 (a membrane protein involved in megalencephalic leukoencephalopathy with subcortical cysts, a type of leukodystrophy) and docks both complexes (GlialCAM-ClC-2 and GlialCAM-Mlc1) at cell-cell junctions (López-Hernández et al., 2011; Jeworutzki et al., 2012; Hoegg-Beiler et al., 2014). Co-expression of GlialCAM with ClC-2 increases currents and almost eliminates the inward rectification, rendering ClC-2 channels nearly constitutively open (Jeworutzki et al., 2012). Disruption of either GlialCAM or Mlc1 affects the expression and localization of ClC-2 (Hoegg-Beiler et al., 2014). Mutations of either GlialCAM or Mcl1 genes lead to megalencephalic leukoencephalopathy, a type of leukodystrophy characterized by early-onset macrocephaly and delayed-onset neurologic deterioration, symptoms comparable to the neurological phenotype of ClC-2 disruption (López-Hernández et al., 2011).

ClC-2 gating is affected by ATP and, like ClC-1, ATP changes the voltage dependence of the common gating. Whole-cell patch clamp recordings show slow activation and deactivation times. Single channel recordings exhibit longer periods of closed states of the common gating when high levels of intracellular ATP are present. This effect, however, does not change the open probability of the channel (Stölting et al., 2013).

#### ClC-2 in Azoospermia and Leukodystrophy

In the testes, tight junctions between Sertoli cells isolate the adluminal compartment of the seminiferous tubules from the blood irrigation (blood–testis barrier). Because of this barrier, maturation and differentiation of spermatogonia into sperm cells require a close physical contact with Sertoli cells that are also responsible for the nourishment of the germ cells during this process. Disruption of ClC-2 function results in transepithelial transport defect in Sertoli cells and subsequent degeneration of male germ cells (azoospermia), as observed in ClC-2 knock-out (KO) mice (Bösl et al., 2001; Bi et al., 2013).

ClC-2 KO mice also develop leukodystrophy (the general term for diseases affecting the growth or maintenance of the white matter), which culminates with gradual development of vacuoles in the myelin sheath of the central nervous system, worsening with age (Blanz et al., 2007). Human patients carrying mutations that disrupt ClC-2 function develop similar leukodystrophy symptoms (Depienne et al., 2013); in one patient, azoospermia was found together with a subclinical leukodystrophy (Di Bella et al., 2014). This was the first case report demonstrating azoospermia and leukodystrophy in a patient with ClC-2 mutation.

#### Other Controversial Physiological Roles

**FIGURE 3:**
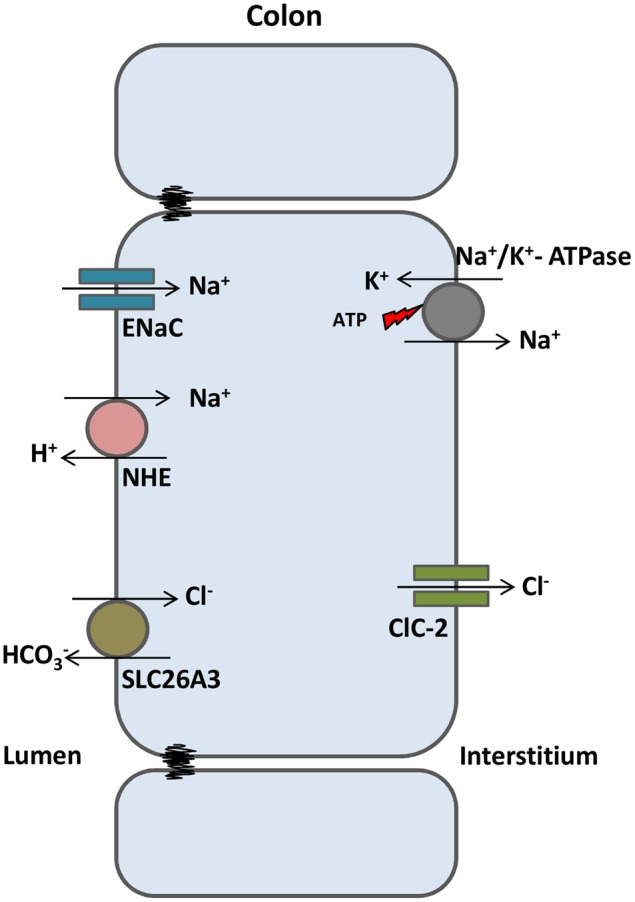
**ClC-2 aids in water absorption in intestinal epithelia.** In colonic enterocytes, chloride absorbed from the intestinal lumen (via SLC26A3 chloride/bicarbonate exchanger) is transported to the interstitium through ClC-2. Sodium enters the cell via ENaC channels or sodium/proton exchangers and is transported to the interstitium through the Na+/K+ ATPase. High NaCl gradient at the interstitium induces osmotic water absorption from the lumen.

ClC-2 is also expressed in epithelial cells of the gastrointestinal tract and lungs. In the past, ClC-2 was proposed to play a role in Cl^-^ efflux at the apical membrane of epithelial cells of these tissues, working as an alternative pathway to CFTR-dependent Cl^-^ secretion. However, the intestinal phenotype observed in CFTR-KO mice was not aggravated in double KO mice, in the absence of both CFTR and ClC-2. Instead, double KO mice survived better than CFTR-KO mice (Zdebik et al., 2004). Later on, it was demonstrated that ClC-2 localizes at the basolateral membrane of enterocytes, facilitating water and salt absorption (**Figure 3**) (Catalán et al., 2004). In the basolateral membrane, ClC-2 is proposed to move Cl^-^ in the opposite direction of CFTR, e.g., moving Cl^-^ from the cell to the interstitium. Loss of ClC-2 in CFTR-KO mice would then increase Cl^-^ concentration inside the cell, facilitating Cl^-^ efflux in the apical compartment by an alternative pathway and compensating for the loss of CFTR from the apical membrane. These and other reports (Catalán et al., 2004; Peña-Münzenmayer et al., 2005) provide convincing data for the basolateral localization of ClC-2 in intestinal epithelia. ClC-2 could play the same role in the lung epithelium, although its precise localization is still not conclusive.

ClC-2 is also expressed in neurons and glial cells, where it is proposed to lower the intracellular concentration of Cl^-^. ClC-2 would be activated after a Cl^-^ influx mediated by hyperpolarizing GABA currents. ClC-2, then, would extrude the excess of intracellular Cl^-^ down to its electrochemical equilibrium helping in the maintenance of a Cl^-^ gradient favorable to cell hyperpolarization by GABA currents (Staley et al., 1996; Földy et al., 2010; Rinke et al., 2010). This theory, however, was questioned by a study using a computational model—based on ClC-2 parameters previously characterized in CA1 pyramidal cells—simulating physiological conditions which showed ClC-2 actually mediating chloride influx, directly reducing cell excitability (Ratté and Prescott, 2011).

The retinal pigment epithelia (RPE) are responsible for forming the blood–organ barrier in the eye, creating the optimal microenvironment for photoreceptor function. Loss of retinal photoreceptors induces retinal degeneration. Loss of ClC-2 function has been proposed to affect transepithelial transport in the RPE by disrupting microenvironment ion homeostasis, resulting in photoreceptor degeneration (Bösl et al., 2001; Bi et al., 2013). Studies on ClC-2 KO mice revealed retinal degeneration, indicating an important role for this channel in RPE. This degenerative phenotype suggests the disruption of ion homeostasis in this tissue (Bösl et al., 2001).

Previously, several other functions were thought to be assigned to ClC-2. Suggested roles in gastric acid secretion (Sherry et al., 2001) and lung development (Murray et al., 1995) were supported neither by experimental data nor by ClC-2 KO mice phenotype. A role in epilepsy was also considered, but after the retraction of a widely cited paper correlating ClC-2 mutations to idiopathic generalized epilepsy, there is no credible evidence for a ClC-2 role in human epilepsy. This is consistent with the lack of seizures observed in ClC-2 KO mice (Bösl et al., 2001; Blanz et al., 2007).

### ClC-Ka and ClC-Kb: Largely Open ClC Channels That Require a β-Subunit

ClC-Ka and ClC-Kb (-K1 and -K2 in rodents) are two closely related ClC channels (around 90% identical) (Adachi et al., 1994; Kieferle et al., 1994), expressed almost entirely in nephrons and in the stria vascularis of the inner ear (Uchida et al., 1995; Estévez et al., 2001). Different from the other mammalian ClC channels, the two ClC-K isoforms lack the ‘gating glutamate,’ displaying halide selectively sequence of Br^-^ > Cl^-^ > I^-^ (Adachi et al., 1994). ClC-K channels have only a slight voltage-dependent gating and hence are open over a broad voltage range (Estévez et al., 2001; Stauber et al., 2012). The first heterologously expressed ClC-Ka and -Kb channels (and also the mice ClC-K2) failed to display any conductance (Kieferle et al., 1994), which raises questions about the necessity of a β-subunit, given that immunohistochemistry (Uchida et al., 1995; Vandewalle et al., 1997) and disease-causing mutations (Simon et al., 1997) clearly indicate their participation in transepithelial salt transport.

Barttin, a 40 kDa and 320-residue protein containing two transmembrane domains and a long intracellular C-terminal domain (Birkenhäger et al., 2001) was identified as the required accessory protein for human ClC-K proteins to be functional (Estévez et al., 2001). Barttin is essential for channel function, stability, and trafficking to the correct membrane area within the cell (Estévez et al., 2001; Waldegger et al., 2002; Scholl et al., 2006). The transmembrane region of barttin is important for its association with ClC-K proteins as well as trafficking to the plasma membrane, whilst the initial part of the C-terminal domain is essential for channel conductance activation (Scholl et al., 2006).

To date, there has been little investigation of interactions between barttin and ClC-K proteins, but two helices of ClC-K are proposed to interact with the transmembrane domain of barttin (Tajima et al., 2007). To further investigate the molecular determinants of barttin/ClC-K interactions, Wojciechowski et al. (2015) used tryptophan scanning mutagenesis to identify amino acids in the transmembrane domains of barttin essential for ClC-K function. Taking into account only normally expressing barttin mutants (some mutants were misfolded or had low expression), substitution of six amino acids (three in each of the transmembrane domains) affected ClC-K/barttin trafficking to the membrane. In contrast, several mutations directly affected ClC-K function. ClC-K currents were abolished when co-expressed with 12 barttin mutants (nine at the first and three at the second transmembrane domains) while two tryptophan insertions at the second transmembrane caused reduced current amplitudes. As most inactivating mutants had tryptophan insertions at the first transmembrane domain, the authors suggest that this domain is critical for activation of ClC-K channels (Wojciechowski et al., 2015).

Co-expressed ClC-K/barttin channels display a very high Cl^-^ conductance (∼40 pS compared to ∼1 pS for ClC-1 and ∼3 pS for ClC-2), modulated by extracellular pH and Ca^+2^ concentration; function is inhibited by H^+^ and activated by Ca^+2^. However, the physiological importance of these modulations are still unclear. ClC-K/barttin localizes at the basolateral membranes of both the thin and thick ascending limbs of Henle’s loop, and in marginal cells of the stria vascularis of the inner ear (Estévez et al., 2001). ClC-K1 was also found in the apical membrane of the thin ascending limb of Henle’s loop (Uchida et al., 1995).

#### ClC-K in Renal Salt Loss and Deafness

**FIGURE 4:**
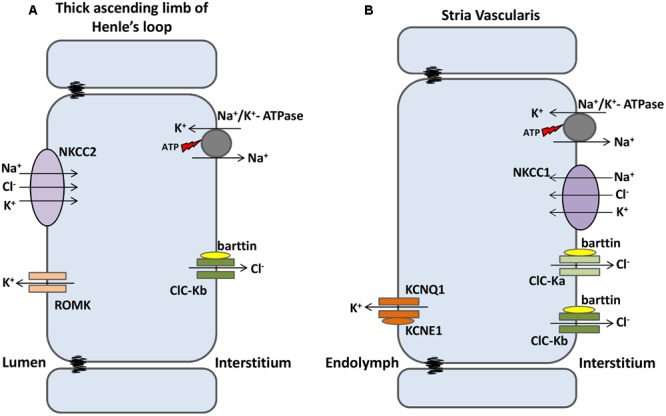
**ClC-K channels are expressed in kidney and inner ear. (A)** At the nephrons, luminal NKCC2 transporters build up Na^+^, K^+^ and Cl^-^ into the cells. K^+^ flows back to the lumen through ROMK1 channels; Na^+^ and Cl^-^ are reabsorbed to the bloodstream separately through Na+/K+ ATPase and ClC-Kb channels, respectively. **(B)** In the Stria Vascularis, Na^+^, K^+^ and Cl^-^ are transported into the cells by basolateral NKCC1 transporters. Na^+^ and Cl^-^ are recycled back to the interstitium by Na+/K+ ATPase and both ClC-Ks isomers, respectively. K^+^ flows through KCNQ1/KCNE1 channels and accumulates into the endolymph, a condition required for sensory transduction in inner hair cells.

ClC-Kb/barttin is mainly expressed in basolateral membranes of the thick ascending limb of Henle’s loop, where it is involved in the reabsorption of salt and, consequently, water (Uchida et al., 1995). In this part of the nephron, the Na^+^ electrochemical gradient (created by basolateral Na^+^/K^+^ pump) drives the secondary active transport of NKCC2 (present in the apical membrane), accumulating Na^+^, Cl^-^, and K^+^ into the cell. K^+^ is extruded back to the lumen through ROMK K^+^ channels (also present in the apical membrane), whereas Na^+^ and Cl^-^ are reabsorbed by the interstitial fluid through the Na^+^/K^+^ pump and ClC-Kb channels, respectively. Thus, the end product of this system is the reabsorption of NaCl into the blood stream (**Figure 4A**).

In the inner ear, both ClC-K isomers are expressed in the basolateral membrane of marginal cells of the stria vascularis. This multilayered epithelium is responsible for both the high concentration of K^+^ and the positive potential (about 100 mV higher than normal extracellular fluids) of the endolymph of the scala media, both of which are important properties for hearing. In marginal cells—the more apical layer in the stria vascularis—Na^+^/K^+^ pumps and NKCC1 transporters build up K^+^ and Cl^-^ inside the cells. ClC-K/barttin channels recycle Cl^-^ back to the interstitial fluid, while apical KCNQ1/KCNE1 K^+^ channels secrete the excess of potassium ions into the endolymph (**Figure 4B**) (Rickheit et al., 2008).

In agreement with the transport models involving ClC-K/barttin channels, mutations in the gene encoding ClC-Kb cause salt-losing Bartter syndrome type III (Simon et al., 1997), characterized by hypokalemia, metabolic alkalosis and secondary hyperaldosteronism with normal or low blood pressure (Andrini et al., 2015). Mutations in the gene encoding barttin cause Bartter syndrome type IV that combines the salt waste with congenital deafness, since both ClC-K proteins are non-functional in the absence of barttin (Birkenhäger et al., 2001). When disruption occurs in only one of the ClC-K channels, as it does in ClC-Kb mutations in Bartter type III, hearing is preserved; the other isomer channel still provides the necessary Cl^-^ recycling. Deafness occurs only on disruption of both ClC-K channels or upon disruption of barttin (Birkenhäger et al., 2001; Schlingmann et al., 2004).

Although there are no reports of patients presenting mutations only in ClC-Ka, two patients presenting symptoms similar to those accompanying Bartter syndrome type IV—severe renal salt loss and sensorineural deafness—were described with loss-of-function mutations in both ClC-K isomers instead of barttin (Schlingmann et al., 2004; Nozu et al., 2008).

#### ClC-K Involvement in Cardiovascular Diseases

Polymorphisms in ClC-Ka and -Kb genes were described, and their relationship with cardiovascular diseases was analyzed. ClC-Kb gene polymorphism T481S increases currents in heterologously expressed channels by approximately 20-fold (Jeck et al., 2004). This may lead to increased salt reabsorption in the thick ascending limb of Henle’s loop, suggesting a possible connection with hypertension. However, several cohort studies found discrepant results, and a link between this activating polymorphism and hypertension is still lacking (Jeck et al., 2004; Speirs et al., 2005; Fava et al., 2007; Sile et al., 2009). One frequent polymorphism in the ClC-Ka gene (R83G) was linked to heart failure. R83G was reported to reduce ClC-Ka currents by about 50%, and was statistically associated with heart failure in three independent Caucasian cohorts (Cappola et al., 2011). However, a functional link between this half-loss-of-function polymorphism and heart failure is still not established.

ClC-K/barttin channels are promising candidates for therapeutic drugs. As ClC-Kb is involved in salt and water reabsorption in the thick ascending limb of Henle’s loop, drugs blocking ClC-Kb/barttin function could reduce renal salt and water reabsorption, which would decrease blood volume and consequently reduce blood pressure. In the inner ear, hearing depends on the depolarization of mechanosensitive hair cells. Different from other excitable cells that use Na^+^ currents for depolarization, depolarization of hair cells is mediated by K^+^ influx.

Drugs capable of increasing ClC-K/barttin function in the stria vascularis would increase endolymph K^+^ concentration, and therefore could be used to treat hearing disorders. However, due to expression of ClC-K/barttin channels in both the kidney and inner ear, it will be difficult to develop specific drugs without undesirable side effects. Recently, while testing new benzofuran derivatives designed to block ClC-K function, Liantonio et al. (2016) described the most potent and selective ClC-K blocker discovered to date (SRA-36). This compound is able to inhibit not only wild-type channels, but also the Cl^-^ currents of polymorphic ClC-K channels associated with hypertension (Liantonio et al., 2016). Although several studies have made significant progress on the identification of compounds modulating ClC-K channel function (Liantonio et al., 2004, 2006, 2016; Picollo et al., 2004), there are not yet therapeutic drugs available.

### ClC-5: A ClC Exchanger of Early Endosomes

**FIGURE 5:**
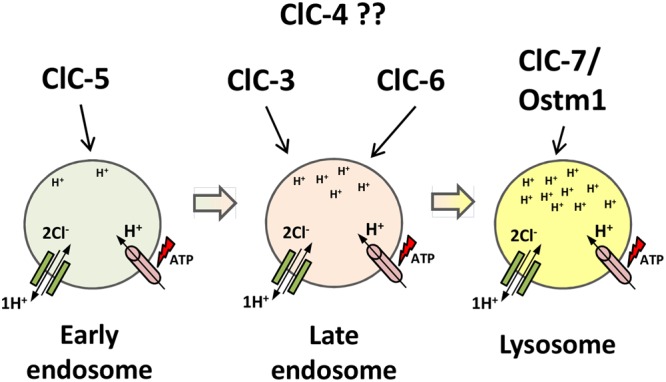
**Proposed localization of intracellular CLC exchangers to the endosomal/lysosomal pathway.** ClC-5 localizes to earlier compartments of the pathway; ClC-3 and ClC-6 localize to later endosome compartments; ClC-7/Ostm1 localizes to lysosomes, the most acidic compartment. ClC-4 localization is still unclear. The ATP-proton pump (pink) acidifies the compartments, increasing protons concentration down the pathway. ClC exchangers (green) provide the shunt current in early endosomal compartments and accumulate chloride in lysosomes.

ClC-5 is the most well-studied member of the second branch of the ClC family. It was identified independently by linkage analysis of patients with Dent’s disease (Fisher et al., 1994) and by homology cloning (Steinmeyer et al., 1995). Unlike ClC-3 and ClC-4, ClC-5 has a more restricted tissue distribution, localizing mostly in renal and intestinal epithelia (Steinmeyer et al., 1995; Vandewalle et al., 2001). In the kidney, ClC-5 is mostly expressed in acid-transporting intercalated cells in distal nephron and in PTCs (Günther et al., 1998). In PTCs, ClC-5 is co-localized with V-type H^+^-ATPase at early and recycling endosomes, with only a small amount found at the surface membrane of brush cells (Günther et al., 1998). In intestinal epithelia, ClC-5 also co-localizes with the proton ATPase in apical endosomes (**Figure 5**) (Vandewalle et al., 2001).

Although it is mainly localized in apical endosomes, a reasonable amount of ClC-5 can be found at the cell surface upon heterologous overexpression, where it can be biophysically analyzed (Steinmeyer et al., 1995; Friedrich et al., 1999). ClC-5 is a 2Cl^-^/1H^+^ exchanger (Picollo and Pusch, 2005; Scheel et al., 2005) with an anion conductance sequence of Cl^-^ > Br^-^ > I^-^, and displays strong outwardly rectifying current that is decreased by low extracellular pH, similar to the closely related ClC-4 (Steinmeyer et al., 1995; Friedrich et al., 1999). Similar to other ClC exchangers, neutralization of the ‘gating glutamate’ results in uncoupled Cl^-^ passive conductance and eliminates voltage dependence (Picollo and Pusch, 2005; Scheel et al., 2005). Interestingly, however, the neutralization of Glu_int_—the putative intracellular proton acceptor—results in disruption of both Cl^-^ and H^+^ transport in ClC-5, creating a transport-deficient protein. That behavior differs from the prokaryotic exchanger ecClC, which exhibits Cl^-^ passive conductance upon either ‘gating glutamate’ or ‘proton glutamate’ mutations (Zdebik et al., 2008). Grieschat and Alekov (2012) found similar results by neutralizing either Glu_ext_ or Glu_int_.

When ClC-5 is expressed heterologously, replacement of extracellular Cl^-^ with SCN^-^ leads to uncoupling of anion transport but does not affect proton transport. The change from extracellular Cl^-^ to SCN^-^ led to increased current amplitudes, and this effect was ∼4-fold higher when intracellular pH was reduced. The effect of intracellular protons was suggested to be related to the protonation of Glu_ext_. Neutralizing either Glu_ext_ or Glu_int_ (E211C; E268C) eliminated the effect of low pH. With neutralization of Glu_ext_ the cysteine side chains are not available for protonation. With neutralization of Glu_int_ cysteine is unable to supply protons to Glu_ext_ (Grieschat and Alekov, 2012). In the case of the E268C mutant, transport was restored to wild-type levels after reaction of a negatively charged and protonable MTSES compound with C268, indicating that the ability of Glu_int_ to protonate Glu_ext_ regulates transport probability in ClC-5 (Grieschat and Alekov, 2012).

The cytoplasmic domain of ClC-5 was found to bind adenosine nucleotides in a site located between the CBS domains. As binding of AMP, ADP or ATP occurs with similar affinities, the physiological role of nucleotide binding remains unclear (Meyer et al., 2007). Also, in the region between its two CBS domains, ClC-5 carries a PY-motif known to bind WW-domains of ubiquitin ligases (Schwake et al., 2001). Point mutations that eliminate the PY-motif of ClC-5 double the currents and increase cell surface localization upon heterologous expression (Schwake et al., 2001). However, knock-in mice with a point mutation disrupting the PY-motif lack any of the effects observed *in vitro* (Rickheit et al., 2010).

#### ClC-5 and Dent’s Disease

Dent’s disease is a rare X-linked kidney disorder associated with low molecular weight proteinuria, hyperphosphaturia, hypercalciuria, kidney stones, and nephrocalcinosis (Wrong et al., 1994). After the identification of ClC-5 mutations as the cause of Dent’s disease (Lloyd et al., 1997), more than 100 such mutations were described (Pusch and Zifarelli, 2015). Most mutations in ClC-5 are missense and non-sense mutations, with many of them located at or near the subunit’s interface, resulting in non-functional truncated proteins (Wu et al., 2003; Stauber et al., 2012). Two missense mutations (G212A and E267A) were analyzed regarding their functional consequences. The particular interest in these mutations is explained by their close proximity to Glu_ext_ (E211) and Glu_int_ (E268). Both mutations result in impaired endosomal acidification, however, the causes are distinct. For the G212A mutant, a shift to more depolarizing potentials is the cause of reduced transport, whereas in E267A mutant the inability to transport intracellular protons results in an incomplete transport cycle (Alekov, 2015).

Proximal tubule cells are the main site for re-uptake of low molecular weight proteins from the primary urine filtrated at the glomeruli. The co-localization of ClC-5 and H^+^-ATPase in PTCs, and the loss of low molecular weight proteins in Dent’s disease patients, suggests that ClC-5 might be involved in early tubular endocytosis in nephrons. To better investigate this hypothesis, two ClC-5 KO mice models were independently generated. Both models showed loss of low molecular weight proteins and high levels of retinal- and Vitamin D-binding proteins in the urine—proteins also elevated in the urine of Dent’s disease patients (Wrong et al., 1994), and defective endocytosis in the proximal tubule (Piwon et al., 2000; Wang et al., 2000). Interestingly, only one of the mouse models displayed hypercalciuria and interstitial calcium accumulation (Wang et al., 2000). *In vivo* endocytosis experiments showed that both fluid-phase and receptor-mediated endocytosis were severely reduced, and that the apical expression of NHE3 (Na^+^-H^+^ exchanger) and NaPi-2a (coupled Na^+^-Pi co-transporter) were also reduced, all in a cell-autonomous effect of ClC-5 disruption (Piwon et al., 2000).

Moreover, the protein megalin—an endocytotic receptor responsible for the endocytosis of several proteins and other substances—and its co-receptor cubilin were also decreased in the brush border membrane of PTCs from ClC-5 KO mice (Piwon et al., 2000; Christensen et al., 2003). Although almost all Dent’s disease patients present low molecular weight proteinuria, with values ranging from 100- to over 1000-fold of the normal excretion values, the other clinical features show great variability (Scheinman, 1998; Claverie-Martín et al., 2011).

##### Role of ClC-5 in low weight proteinuria, hyperphosphaturia, and hypercalciuria

Luminal acidification is necessary for proper endosome function (Clague et al., 1994) and ClC-5 was thought to provide the electrical shunt necessary for the acidification of endosomes by proton pumps (Piwon et al., 2000). Cl^-^ ions transported by ClC-5 provide the negative charge necessary for neutralization of protons accumulating at the lumen of endosomes by the proton pump (the electrical shunt), thus maintaining the acidification process (Hara-Chikuma et al., 2005a). Indeed, ATP-induced acidification in endosomes from ClC-5 KO animals was reduced compared to wild-type animals (Günther et al., 2003; Novarino et al., 2010). Furthermore, endocytosis experiments with cultured PTCs using fluorescent-tagged markers for early/recycling and late endosomes showed a reduction in both acidification and Cl^-^ accumulation in early, but not in late, endosomes (Hara-Chikuma et al., 2005a). A defect in endocytosis was also observed in cultured PTCs from ClC-5 KO mice (Wang et al., 2005).

Processes underlying the other symptoms of Dent’s disease—such as hyperphosphaturia, hypercalciuria, and kidney stones—are more complex. Reduced megalin expression at the brush border membrane of PTCs due to ClC-5 disruption impairs the endocytosis of PTH. Accumulation of PTH at the renal tubules stimulates PTH receptors, which in turn results in degradation and internalization of NaPi-2a transporters, causing a reduction of phosphate re-absorption. Therefore, hypophosphatemia/hyperphosphaturia is observed in both Dent’s disease patients (Claverie-Martín et al., 2011) and ClC-5 KO mice (Piwon et al., 2000). About 30% of Dent’s disease patients display hypophosphatemia due to loss of phosphate in the urine (Claverie-Martín et al., 2011).

Proximal tubule cells are also the main site for vitamin D metabolism, which plays a critical role in Ca^2+^ homeostasis. In these cells, the inactive precursor 25(OH)-VitD_3_ is converted to the active form 1,25(OH)_2_-VitD_3_ by the mitochondrial enzyme 1α-hydroxylase, which is stimulated by PTH (Murayama et al., 1999). Megalin mediates the endocytosis of both active and inactive forms of Vitamin D. Thus, in ClC-5 KO mice, whose lack of ClC-5 results in low megalin expression in the brush border of PTCs and impaired endocytosis, two stimuli may upregulate 1α-hydroxylase expression: (1) overstimulation of PTH receptors, and (2) decreased endocytosis of the active form 1,25(OH)_2_-VitD_3_, as this form represses enzyme transcription (Murayama et al., 1999; Piwon et al., 2000; Günther et al., 2003; Maritzen et al., 2006). Meanwhile, reduced endocytosis of the inactive form 25(OH)-VitD_3_ is also in place, which would cause downregulation of 1α-hydroxylase expression.

**FIGURE 6:**
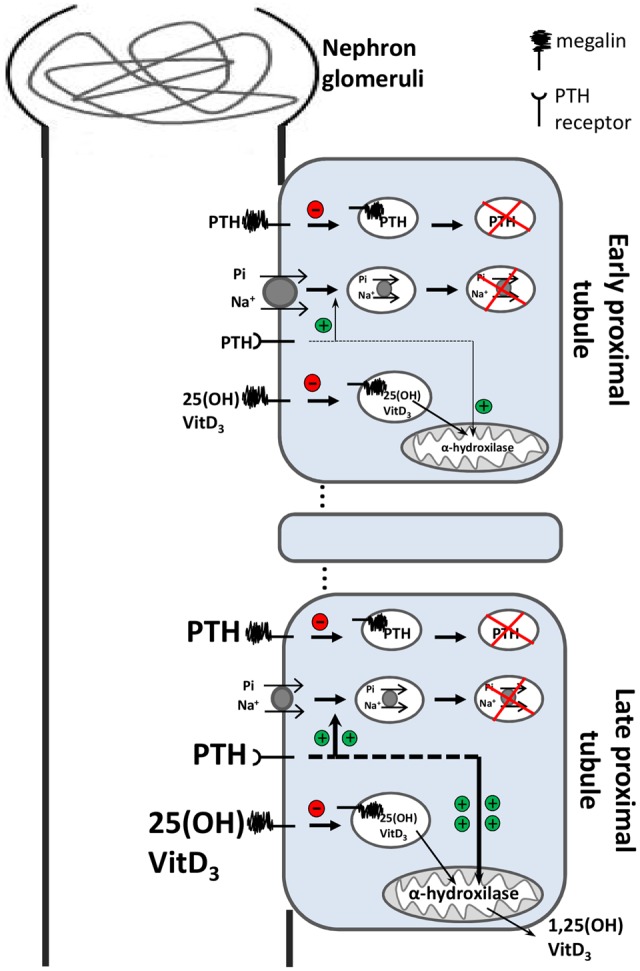
**Simplified model of the effect of impaired endocytosis due to ClC-5 dysfunction in proximal tubular cells.** In the early proximal tubule, PTH and vitamin D3 (both precursor and active molecules) are filtered into the primary urine. Those molecules are generally reabsorbed by megalin-mediated endocytosis and subsequently degraded in lysosomes. The megalin-mediated pathway is severely impaired in ClC-5 dysfunction. Under normal conditions 25(OH)-vitamin D_3_ is metabolized by the mitochondrial enzyme 1α-hydroxylase to the active hormone 1,25(OH)_2_-vitaminD_3_. In the late proximal tubules, accumulation of PTH (due to impaired megalin-mediated endocytosis) overstimulates PTH receptors, which stimulate internalization and degradation of NaPi-2a transporters (green plus), reducing phosphate re-absorption—hyperphosphaturia. Overstimulated PTH receptors also upregulate the transcription of the mitochondrial enzyme 1α-hydroxylase (green plus). As megalin-dependent 25(OH)-vitaminD_3_ endocytosis is impaired, low levels of the precursor vitamin D have access to its converting enzyme. Therefore, the delicate balance between the regulation of 1α-hydroxylase transcription (that cooperates to increase active vitamin D_3_ levels) and the loss of the precursor form of vitamin D_3_ into the urine (preventing its access to the enzyme), determine the outcome of either increased or decreased concentration of active vitamin D_3_ in serum. Because vitamin D3 levels drives the absorption of calcium to the bloodstream, hypercalciuria and kidney stones may or may not develop.

As regulation of 1,25(OH)_2_-VitD_3_ levels is governed by these two opposing mechanisms, it was hypothesized that the balance between precursor levels and those of its converting enzyme will determine the presence—or not—of hypercalciuria (**Figure 6**). If higher levels of 1α-hydroxylase lead to higher levels of 1,25(OH)_2_-VitD_3_ in the serum, more calcium will be absorbed in the intestine; therefore, more calcium will be excreted in the urine, resulting in hypercalciuria and kidney stones. Indeed, patients with Dent’s disease display a high prevalence (∼90%) of hypercalciuria (Claverie-Martín et al., 2011), as well as elevated levels of 1,25(OH)_2_-VitD_3_ (Scheinman, 1998).

Moreover, ClC-5 KO mouse models from Wang et al. (2000) presenting hypercalciuria and renal calcium deposits also displayed high levels of serum 1,25(OH)_2_-VitD_3_ (Wang et al., 2000). In contrast, Piwon et al. (2000) did not identify hypercalciuria in their ClC-5 KO mice, which displayed reduced levels of serum 1,25(OH)_2_-VitD3 (Piwon et al., 2000; Maritzen et al., 2006). There is a lack of reports associating hypercalciuria and calcium deposits with serum levels of Vitamin D. Prospective studies in large cohorts would be a valuable tool in the search for pathophysiological mechanisms underlying Dent’s disease. Dent’s disease is an excellent example of how a primary defect (impaired endocytosis) in a restricted group of cells (PTCs) can lead to a cascade of serious secondary complications (phosphaturia, calciuria, kidney stones, and nephrocalcinosis).

#### ClC-5 As a Cl^-^/H^+^ Exchanger: New Insights on Its Role in Endosomes

ClC proteins are involved in the acidification of both early and late endosomes. Endosomal acidification is a process mediated by active proton influx carried by the H^+^-ATPase. The inward H^+^ movement requires a charge balance, which can be achieved both by outward movement of cations such as K^+^, and by inward movement of anions such as Cl^-^. Extensive experimental data suggest that Cl^-^ is the principal ion providing the electrical shunt for luminal acidification of endosomes (Bae and Verkman, 1990; Al-Awqati, 1995; Grabe and Oster, 2001). After ClC-5 was determined to be a Cl^-^/H^+^ exchanger and not a Cl^-^ channel (Picollo and Pusch, 2005; Scheel et al., 2005), its role as an electrical shunt for proton pumps was questioned; such an exchanger would provide a counter-current of H^+^, opposing the ATP-driving accumulation of protons.

To assess the consequences of this new CIC-5 feature, knock-in mice were generated carrying a point mutation in the ‘gating glutamate’ (E211A) that uncouples Cl^-^ and H^+^ transport, converting ClC-5 to a pure passive Cl^-^ conductor (called ClC-5^unc^; for *unc*oupled) (Novarino et al., 2010). Surprisingly, these mice presented normal renal endosomal acidification, but also an impaired proximal tubular endocytosis similar to that found in ClC-5 KO mice. Two facts suggest that other parameters such as Cl^-^ concentration may play a critical role in endocytosis. First, ClC-5^unc^ mice presented phenotypes similar to the ClC-5 KO group, including hypercalciuria and hyperphosphaturia. Second, PTC endosomes from ClC-5^unc^ showed normal acidification but impaired endocytosis. Recently, a patient with Dent’s disease was identified as carrying a similar mutation in the ‘gating glutamate’ (E211Q) (Sekine et al., 2014). Further support for the role of Cl^-^ concentration in endocytosis comes from mathematical models of simplified vesicles (containing a proton pump, a proton leak and either a Cl^-^/H^+^ exchanger or a Cl^-^ channel) predicting that coupled transport would provide a higher endosomal Cl^-^ concentration than a pure Cl^-^ current (Weinert et al., 2010). Unfortunately, the exact role of ClC-5 coupled Cl^-^/H^+^ transport in early endosomes is still not fully understood.

### ClC-7: A lysosomal ClC Exchanger That Requires a β-Subunit

ClC-7 is another broadly expressed ClC protein. In mouse embryos, ClC-7 was found most prominently expressed in the brain, eyes, spinal cord, and dorsal root and trigeminal ganglia in mouse embryos (Kornak et al., 2001), whereas in adult mice it was found in medulla oblongata, Purkinje cells, PTCs, Sertoli cells, and pancreatic and tracheal epithelia (Kida et al., 2001). ClC-7 localizes mostly in lysosomes (**Figure 5**) (Kornak et al., 2001; Kasper et al., 2005; Poët et al., 2006; Wartosch et al., 2009), but is also found in the ruffled border of osteoclasts (Kornak et al., 2001; Lange et al., 2006). ClC-7 is the only ClC exchanger that requires a β-subunit, Ostm1, for proper function. Ostm1 (osteopetrosis-associated membrane protein 1), a highly glycosylated type 1 transmembrane protein, is essential for stability and transport activity of ClC-7 (Lange et al., 2006; Leisle et al., 2011). Mutations in the Ostm1 gene underlie the spontaneous *gray-lethal* mouse mutant (Chalhoub et al., 2003). Ostm1 and ClC-7 co-localize in lysosomes and in the ruffled border of osteoclasts and maintain a closely dependent relationship, in which protein levels of one are reduced by approximately 95% in the absence of the other (Lange et al., 2006). Moreover, Ostm1 needs to interact with ClC-7 in order to exit the ER and traffic to lysosomes, whereas ClC-7 needs Ostm1 to be stable and functional (Lange et al., 2006; Stauber and Jentsch, 2010). The transmembrane domain of Ostm1 is necessary for ClC-7 trafficking to lysosomes, while the highly glycosylated N-terminus plays a critical role in transport activity of ClC-7 (Leisle et al., 2011).

For many years, the intracellular localization of CLC-7 has hindered the study of its biophysical properties. However, after the identification of a sorting motif localized at the cytosolic N-terminus that directs ClC-7 to lysosomes (Stauber and Jentsch, 2010), point mutations that disrupt this motif allowed partial cell-surface localization of ClC-7 upon heterologous expression, allowing its biophysical characterization (Leisle et al., 2011). ClC-7 shares several characteristics with other ClC exchangers such as the strong outward rectification; anion sequence conductance of Cl^-^ > I^-^; inhibition of activity upon low extracellular pH; and a classical 2Cl^-^/1H^+^ stoichiometry. However, activation and deactivation of ClC-7 are very slow compared to other ClC transporters, allowing for the analysis of tail currents. Tail currents revealed that the exchange process is almost linearly voltage-dependent, and rectification is almost entirely due to a voltage gating (Leisle et al., 2011). Later, slow voltage-dependent activation and deactivation of ClC-7 were assigned to the common gating mechanism (Ludwig et al., 2013). ClC-7 also carries both gating and proton glutamates; mutation of these residues, such as is found in ClC-5, yields a protein displaying a Cl^-^ conductance uncoupled from H^+^ co-transport and a non-functional ClC-7 protein, respectively (Kornak et al., 2001; Leisle et al., 2011).

#### ClC-7 in Osteopetrosis, Retinal Degeneration, and Lysosomal Storage Disease

To study the physiological roles of ClC-7/Ostm1, knock-out mouse models were generated and analyzed. ClC-7 KO mice present short life spans, severe osteopetrosis, retinal degeneration, lysosomal storage disease, and neurodegeneration (Kornak et al., 2001; Kasper et al., 2005). *Gray-lethal* mice (Ostm1 KO) display a very similar phenotype (Chalhoub et al., 2003; Lange et al., 2006), as expected for these two closely functionally related proteins. Interestingly, both ClC-7 KO and Ostm1 KO mice have gray fur in an *agouti* background (in which wild-type mice have brown fur), suggesting a possible role of ClC-7/Ostm1 in melanosomes (Kornak et al., 2001).

**FIGURE 7:**
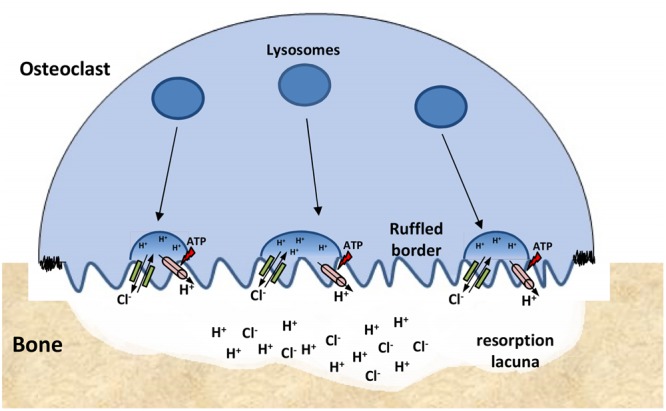
**ClC-7 function at osteoclasts.** Lysosomes are inserted into the ‘ruffled border’ of bone-attached osteoclasts. The resorption lacuna is then acidified by the combined work of proton pumps (pink) and ClC-7/Ostm1 exchangers (green) present in the lysosomes membranes. Low pH conditions are required for the chemical dissolution of inorganic bone material and for the activity of lysosomal enzymes that are secreted into the lacuna.

Loss of function of ClC-7 in osteoclasts results in osteopetrosis, a disease characterized by increased bone radiodensity because of ineffective osteoclast-mediated bone resorption (Shapiro, 1993). The ruffled border of osteoclasts—a membrane domain responsible for acidic digestion of bone tissue—is formed by lysosomal membrane insertion and exocytosis of their content. Acidification of the resorption lacuna—the space between the ruffled border and the bone tissue—is carried by V-type H^+^-ATPase that, similarly to compartments of the endosomal/lysosomal pathway, requires an electrical shunt thought to be performed by ClC-7/Ostm1 (Planells-Cases and Jentsch, 2009; Stauber et al., 2012). In the resorption lacuna, ClC-7/Ostm1 is responsible for the Cl^-^ influx necessary for neutralization (shunting) of protons, which keeps the proton pump functional and maintains the lacuna’s highly acidic environment (**Figure 7**). Indeed, osteoclasts from ClC-7 KO mice displayed underdeveloped ruffled borders and impaired acidification of the resorption lacuna, which causes the osteopetrotic phenotype (Kornak et al., 2001).

After establishment of the link between ClC-7/Ostm1 mutations and osteopetrosis, approximately 50 different human ClC-7 mutations were identified in osteopetrotic patients (for details, see Stauber et al., 2012). Most of these are missense mutations, some of which cause an autosomal dominant form of osteopetrosis that presents less severe symptoms and does not implicate the nervous system (Cleiren et al., 2001; Frattini et al., 2003). Others yield ClC-7/Ostm1 complexes, which are retained in the ER or strongly reduce their ion transport activity (Leisle et al., 2011). However, other missense mutations produce transporters that reach the normal subcellular localization, remaining functional when expressed heterologously in the plasma membrane, and carrying the sorting motif point mutation mentioned above. Curiously, these functional mutants yielded currents with accelerated kinetics of activation and deactivation. Given that patients carrying these mutations present symptoms similar to others with non-functional transporters, the slow gating of ClC-7/Ostm1 is likely physiologically important (Leisle et al., 2011).

In addition to osteopetrosis, ClC-7 KO and *gray-lethal* mice also display retinal and neurodegeneration associated with lysosomal storage (Kornak et al., 2001; Kasper et al., 2005; Lange et al., 2006). Retinal degeneration leads to blindness about 4 weeks after birth (Kornak et al., 2001; Lange et al., 2006). Although previously the blindness was believed to be the result of osteopetrotic narrowing of the optic nerve canal (Steward, 2003), retinal degeneration is a direct effect of disruption of ClC-7 or Ostm1 at retinal neurons (Kasper et al., 2005). Neurodegeneration is the probable cause of death of ClC-7 KO mice at approximately 6 weeks. They present neuronal cell loss, particularly in the hippocampus and cerebral cortex, as well as lysosomal storage material scattered throughout the neuronal somata (Kasper et al., 2005). Neurodegeneration and lysosomal storage disease are cell-autonomous effects of disruption of ClC-7 as demonstrated by tissue-specific ClC-7 KO mice. In this study, PTCs and neurons lacking or expressing ClC-7 were compared within the same environment; only cells devoid of ClC-7 displayed lysosomal disease (Wartosch et al., 2009).

#### New Insights on the Role of C1C-7 in Lysosomes

In correlation with ClC-7 function in osteoclasts and ClC-3 to ClC-5 roles in acidification of their respective compartments (Mohammad-Panah et al., 2003; Hara-Chikuma et al., 2005a,b), the lysosomal storage phenotype was first proposed to result from impaired acidification. However, ratiometric measurements showed that ClC-7/Ostm1 does not play a role in lysosomal acidification; that is, lysosomes from ClC-7 KO mice display normal steady-state pH (Kasper et al., 2005; Lange et al., 2006; Weinert et al., 2010). The counter-charge conductance suggested to neutralize H^+^-ATPase currents in this case seems to be provided by a lysosomal cation efflux (Steinberg et al., 2010).

These intriguing results concerning the function of ClC-7 in lysosomes led to the generation of two other ClC-7 mice models, the ClC-7^unc^ mice (Weinert et al., 2010)—generated similarly to the ClC-5^unc^ mice, by a ‘gating glutamate’ mutation that yields uncoupled Cl^-^ pure conductance—and the ClC-7^td^ mice (Weinert et al., 2014), generated by a mutation in the ‘proton glutamate’ that abolishes transport (td: transport deficient). Much like ClC-7 KO mice, both new models displayed normal lysosomal pH and reduced Cl^-^ concentration. However, although ClC-7^unc^ mice presented neuronal cell loss and lysosomal storage disease similar to the ClC-KO mice, the osteopetrotic phenotype was partially rescued by the presence of pure Cl^-^ currents (Weinert et al., 2010). Meanwhile, ClC-7^td^ mice displayed similar osteopetrotic phenotype to ClC-7 KO mice, but a delayed and less severe neurodegeneration (Weinert et al., 2014).

Surprisingly, unlike ClC-7 KO and Ostm1 KO mice, the two new models presented normal *agouti* brown fur color (Weinert et al., 2014). Taken together, these results suggest that the physical presence of the non-functioning ClC-7^td^ alone was sufficient to rescue the normal fur pigmentation and alleviate neurodegeneration, whereas ClC-7^unc^ Cl^-^ currents had a positive effect in partially rescued osteopetrosis and normal fur pigmentation, but a negative effect in neurodegeneration (Weinert et al., 2014). Therefore, normal fur pigmentation seems to require only the presence of ClC-7/Ostm1 complex, whereas lysosomes and osteoclasts require fully functional Cl^-^/H^+^ exchangers in order to function properly. The precise role of ClC-7 in lysosomes is still obscure, as is the previously discussed role of ClC-5 in endosomes. More work is necessary to better understand the role of coupled Cl^-^/H^+^ transport in the endosomal/lysosomal pathway.

### ClCs with Uncertain Physiological Function

The last sections of this review focuses on ClC-3, -4, and -6, which have uncertain physiological functions. Although mutations or dysfunction of these ClCs have not been linked directly to specific human diseases, mouse models have demonstrated important phenotypes that are worth discussing here.

#### ClC-3: Retinal and Brain Degeneration?

ClC-3 is a broadly expressed intracellular ClC protein with controversial biophysical and physiological characteristics. Several mutually conflicting Cl^-^ currents have been attributed to ClC-3. First, a slightly outwardly rectifying Cl^-^ current inhibited by PKC (Kawasaki et al., 1994); second, Cl^-^ currents inhibited by intracellular Ca^+2^ (Kawasaki et al., 1995), followed by swelling-activated Cl^-^ currents, also known as VRACs (Duan et al., 1997). The role of VRACs was not confirmed by data from three independently generated ClC-3 KO mice (Stobrawa et al., 2001; Dickerson et al., 2002; Yoshikawa et al., 2002), as swelling-activated Cl^-^ currents were unaffected in the hepatocytes, salivary acinar cells and cardiomyocytes (Arreola et al., 2002; Gong et al., 2004). Moreover, a recent report identified LRRC8 proteins as essential components of VRACs, as their disruption abolishes VRAC currents (Voss et al., 2014).

ClC-3 presents very low cell surface expression, even when heterologously overexpressed, which has hindered a thorough analysis of its biophysical properties. Nevertheless, in some studies, small but strongly outwardly rectifying Cl^-^ currents were found (Li et al., 2002; Matsuda et al., 2008; Guzman et al., 2013), similar to those reported for ClC-4 and ClC-5 (Steinmeyer et al., 1995; Friedrich et al., 1999). Moreover, because of its substantial sequence identity—approximately 80% with ClC-4 and ClC-5, which are well established ClC exchangers—and the presence of the conserved ‘gating glutamate,’ which mutation abolish rectification as in other ClCs, ClC-3 is most likely a voltage dependent Cl^-^/H^+^ exchanger (Li et al., 2002; Guzman et al., 2013).

ClC-3 is expressed in most tissues, including the brain, retina, adrenal gland, pancreas, intestines, epididymis, kidney, liver, skeletal muscle, and heart (Kawasaki et al., 1994; Stobrawa et al., 2001; Suzuki et al., 2006; Maritzen et al., 2008). It mainly resides in endosomes, where it was found co-localized with ClC-4 and ClC-5, and also with both early and late endosomal markers (**Figure 5**) (Suzuki et al., 2006). CLC-3 is also found in synaptic vesicles (Stobrawa et al., 2001; Salazar et al., 2004) and synaptic-like micro vesicles (Salazar et al., 2004; Maritzen et al., 2008). Three different ClC-3 KO mouse lines displayed similar phenotypes of severe degeneration of the retina and brain, with prominent effects in the hippocampus (Stobrawa et al., 2001; Dickerson et al., 2002; Yoshikawa et al., 2002). In one model, signs of lysosomal storage disease were observed (Yoshikawa et al., 2002), but these effects were much weaker than those found in ClC-6 and ClC-7 KO mice (Poët et al., 2006; Kasper et al., 2005). The mechanism by which ClC-3 causes neurodegeneration is still unclear.

Although ClC-3 was thought to provide the electrical shunt for acidification of intracellular compartments like the other ClC exchangers, its role in endosomes and synaptic vesicles is still controversial. In ClC-3 KO mice, acidification and Cl^-^ accumulation were reduced in early and late endosomes (Hara-Chikuma et al., 2005b) and synaptic vesicles showed less efficient acidification *in vitro* (Stobrawa et al., 2001). Synaptic vesicles from ClC-3 KO mice exhibit reduced glutamate uptake, but this feature has been ascribed to diminished levels of the vesicular glutamate transporter VGLUT1 (Stobrawa et al., 2001). In another study, evidence against ClC-3’s role in acidifying synaptic vesicles was reported (Schenck et al., 2009). The fact that VGLUT1 KO mice display no chloride-dependent acidification of synaptic vesicles, and very little expression of CLC-3 in synapse vesicles, led the authors to suggest that VGLUT1 represents the major Cl^-^ conductance pathway in synaptic vesicles (Schenck et al., 2009). Therefore, the reduced acidification of synaptic vesicle reported in ClC-3 KO mice (Stobrawa et al., 2001) was attributed to reduced levels of VGLUT1, which itself most likely resulted from severe neurodegeneration.

To avoid the effects of neurodegeneration on VGLUT1 levels, Guzman et al. (2014) used cultured hippocampal neurons to analyze ClC-3 disruption in synaptic vesicles. The authors observed enlargement of synaptic vesicles and increased glutamate content in cells lacking ClC-3. The probability of vesicle fusion and release of its content was also increased in those cells, indicating that exaggerated release of glutamate in the synaptic cleft contributes to neurodegeneration in ClC-3 KO mice (Guzman et al., 2014).

Recently, reduced levels of ClC-3 were found in patients with inflammatory bowel disease (IBD) and from mice treated with dextran sulfate sodium (DSS) to induce colitis and mimic IBD. ClC-3 KO mice were more susceptible to DSS-induced colitis with no signs of recovery after treatment. Lack of ClC-3 provoked apoptosis of intestinal epithelial cells, causing disruption of the epithelial barrier and bacterial invasion. Thus, the authors defend the involvement of ClC-3 in IBDs pathogenesis (Huang et al., 2014). Another study points to ClC-3’s involvement atherosclerosis. In an ApoE null mice background, further disruption of ClC-3 reduced the size of atherosclerotic lesions present in the aorta. The authors suggest that ClC-3 insufficiency disrupts scavenger receptor SR-A expression (via JNK/p38 MAPK) and foam cell formation, leading to reduction/inhibition of atherosclerotic lesions (Tao et al., 2015). The protective effect of ClC-3 deficiency was again addressed in endothelial progenitor cells (EPCs). Angiotensin II-induced apoptosis of EPCs was remarkably reduced in ClC-3 KO mice. This inhibition was attributed to suppressed levels of reactive oxygen species and NADPH oxidase activity—direct effects of angiotensin-II—that are suppressed by ClC-3 disruption (Liu et al., 2013).

Despite ClC-3 diversity in different cell types, phenotypes present in ClC-3 KO mice strongly suggest that ClC-3 is important for neurotransmission in the CNS. Three different splicing variants of ClC-3 (ClC-3a, ClC-3b, and ClC-3c) were described in the brain with different subcellular localization but similar transport function (Guzman et al., 2015). Another splicing variant, ClC-3d, was described in mouse livers as displaying different localizations but identical transport properties (Okada et al., 2014). The number of splicing variants with different subcellular localizations might explain the diversity of functions ascribed to ClC-3; the study of these isoforms could be a promising direction for further study of the precise function and localization of ClC-3 proteins, which could provide an explanation for the phenotypes described in KO mice.

#### ClC-4: A Cl^-^/H^+^ Exchanger with Unclear Physiological Function

ClC-4 is a broadly expressed ClC exchanger found in various tissues which differ between species. It is found mostly in the muscles, brain, and heart of humans (van Slegtenhorst et al., 1994); in the liver and brain, heart, muscles, spleen, and kidneys of rats (Jentsch et al., 1995); and in the brain, intestines, and kidneys of mice (Mohammad-Panah et al., 2003). Interestingly, the gene encoding ClC-4 is localized on chromosome 7 in inbred laboratory mice, but in humans and rats, the gene resides on the X chromosome (Rugarli et al., 1995). This may partially explain the variety and species-specificity of expression patterns.

ClC-4 localizes mainly at endosomes’ membranes; upon heterologous overexpression, a small portion is also found within the plasma membrane (Mohammad-Panah et al., 2003; Suzuki et al., 2006). Like other members of this family, the cell surface localization allows for a better analysis of its biophysical properties. ClC-4 yields a strongly outwardly rectifying current that is inhibited by low extracellular pH; to date, the physiological relevance of pH regulation remains unclear. ClC-4 is a Cl^-^/H^+^ exchanger with anion conductance sequence of Cl^-^ > Br^-^ > I^-^, similar to other intracellular ClCs. Mutation of the ‘gating glutamate’ strongly changes rectification and converts the exchanger into a passive Cl^-^ conductor, highlighting the importance of this residue in coupling proton to Cl^-^ transport (Friedrich et al., 1999; Picollo and Pusch, 2005; Scheel et al., 2005).

Alekov and Fahlke (2009) have shown that ClC-4 proteins, when exposed to different types of anions in the extracellular buffer, can display a phenomenon called ‘slippage,’ where the transporter behaves as a channel rather than an obligatory exchanger. High extracellular SCN^-^ cause increased current amplitudes and uncouple H^+^ transport rendering ClC-4 channel-like transport with reduced H^+^ currents and biophysical properties similar to other well-characterized ClC channels. Restoring extracellular Cl^-^ rescues Cl^-^/H^+^ exchange. In ClC-5, extracellular SCN^-^ uncouples transport but does not affect proton transport (Grieschat and Alekov, 2012), an apparent isoform-specific effect. Thus, ClC-4 is suggested to function as a channel or exchanger depending on the extracellular anion (Alekov and Fahlke, 2009).

Currently, there is little scientific consensus regarding the sub-cellular localization of ClC-4 (**Figure 5**). On those studies, ClC-4 was found in sub-apical vesicles of proximal tubule epithelium (Mohammad-Panah et al., 2003); in intracellular compartments of HEK 293 cells co-localizing with ClC-3 and ClC-5 (Suzuki et al., 2006); and in the endoplasmic reticulum (Okkenhaug et al., 2006). However, none of the immunohistochemistry studies performed thus far have used cells from ClC-4 KO mice as a negative control to confirm their data.

ClC-4 was suggested to facilitate endosomal acidification by working as the electrical shunt for proton accumulation mediated by the proton pump. However, ClC-4 KO mice do not display any obvious abnormal phenotypes (Rickheit et al., 2010). Although ClC-4 trafficking is similar to ClC-5, they do not appear to perform similar physiological functions (Mohammad-Panah et al., 2003). The additional disruption of ClC-4 in ClC-5 KO mice did not aggravate the impaired endocytosis phenotype in PTCs (Rickheit et al., 2010).

One naturally occurring mutation (G544R), found in a patient with severe epilepsy and delayed development, nearly abolished ClC-4 currents when expressed heterologously (Veeramah et al., 2013). Hu et al. (2015), analyzing X-linked intellectual disabilities, identified five different mutations in the ClC-4 gene in five families. Currents of ClC-4 proteins carrying each of these mutations were much smaller or even absent compared to wild-type ClC-4. Moreover, C1C-4 depletion in cultured hippocampal neurons, affected neuronal differentiation; the cells displayed a 30% reduction of neuritic outgrowth and branching (Hu et al., 2015). Additional studies using specific antibodies and appropriate KO controls are necessary to further understand ClC-4 physiological function in specific cell compartments, determine precise sub-cellular localization, and investigate possible roles in human diseases.

#### ClC-6: Mild Lysosomal Storage Disease?

ClC-6 shares approximately 45% of its sequence identity with ClC-7; together, they form the third branch of the ClC protein family. Like other ClC exchangers, ClC-6 localizes at membranes of the endosomal/lysosomal pathway (**Figure 5**) (Poët et al., 2006). ClC-6 mRNA was found in several tissues (Brandt and Jentsch, 1995), but the expressed ClC-6 protein is found almost exclusively in the nervous system (Poët et al., 2006). First attempts to record ClC-6 currents by heterologous expression were frustrated by its late endosomal localization (Brandt and Jentsch, 1995; Buyse et al., 1998), and biophysical characterization only became possible when GFP-tagged ClC-6 proteins were expressed in the plasma membrane (Neagoe et al., 2010). ClC-6 mediates outwardly rectifying currents that are reduced by extracellular acidification, as in other ClC-exchangers. Mutation in the ‘gating glutamate’ also disrupts rectification, turning ClC-6 into a passive Cl^-^ conduit (Neagoe et al., 2010).

Knock-out controlled immunohistochemistry studies have shown that native ClC-6 localizes predominantly at late endosomes of neurons *in situ* (Poët et al., 2006) and in cultured cells (Ignoul et al., 2007), whereas in heterologous expression it is also found co-localized with early and late endosomal markers (Suzuki et al., 2006; Stauber and Jentsch, 2010).

ClC-6 KO mice present no apparent abnormal phenotypes, with normal life span and weight. However, late in life (>3 months old), the mice display a peculiar form of lysosomal storage disease, with deposits found in central and peripheral neurons (Poët et al., 2006). Different from ClC-7 KO mice, in which such deposits are localized all over the neuronal soma and the disease progression is much more aggressive, deposits in ClC-6 KO neurons are mainly localized at initial axon segments and the disease progresses very slowly (Poët et al., 2006; Pressey et al., 2010). Moreover, the absence of ClC-6 in hippocampal neurons does not affect lysosomal steady-state pH (Poët et al., 2006). Deposits found in ClC-6 KO mice tested positive for markers typically found in neuronal ceroid lipofuscinosis (NCL), a lysosomal storage disease. The authors therefore proposed ClC-6 gene as a candidate for mild forms of NCL, but did not find convincing association upon analysis of 75 NCL patients (Poët et al., 2006).

In general, neuropathology in ClC-6 KO mice is much milder than in ClC-3 and ClC-7 KO mice. They show no vision impairment, and little neuronal cell loss and microglial activation (Poët et al., 2006; Pressey et al., 2010). ClC-6 KO mice also demonstrate reduced pain sensitivity, correlated with an impairment of dorsal root ganglion neuronal function due to dramatic lysosomal storage accumulation (Poët et al., 2006). After all, like ClC-3 and ClC-4, ClC-6 is another ClC exchanger whose physiological role is poorly understood at present.

## Conclusion

Cl^-^ ion transport has risen from obscurity to become a vibrant and exciting field in ion transport research. Within this field, ClC proteins are a particularly intriguing family of anion channels and transporters involved in several important physiological functions. Twenty-five years after the discovery of its first member (ClC-0), and following enormous efforts to study their biological aspects, many questions about the structure, function, and pathophysiological roles of ClCs have been answered, but an equally high number of new and, so far, unsolved questions have emerged. For instance, the precise localization of ClC-K channels in the thin limb of the loop of Henle in the kidney and its function in intercalated cells are still unknown. Future research topics of particular interest include a better understanding of the relationship between α- and β-subunits, and of the physiological role of β-subunits by themselves.

Phenotypes of mouse models have linked ClC protein function and dysfunction with inherited human genetic diseases. Myotonia congenita, leukodystrophy, Bartter syndrome, Dent’s disease, and osteopetrosis/retinal degeneration/lysosomal storage disease have well-established association with loss-of-function of ClC1, ClC-2, ClC-K/Barttin, ClC-5 and ClC-7/Ostm1, respectively. However, many aspects of these diseases’ molecular origins remain obscure.

Useful tools to increase our knowledge about the molecular basis of ClC-related diseases would include the development of small molecules able to specifically block or activate ClC proteins. Unfortunately, currently available compounds targeting ClC proteins are few and far between, and they lack specificity. The role of intracellular ClC exchangers in the endosomal/lysosomal pathway is not completely established. Acidification and Cl^-^ accumulation seem not to be the only functions of ClC exchangers in these compartments. Interactions with other cell proteins—and not only transport activity of ClC exchangers—seem to play a role in endosome/lysosome homeostasis, as revealed by the rescue of some pathological phenotypes in KO mice upon the expression of non-functional ClC-7 proteins. Some phenotypes displayed by ClC-3 and ClC-6 KO mice could not be correlated with the physiological roles so far assigned to ClC-3 and ClC-6. However, this may be only a matter of time; recently, leukodystrophy and azoospermia—typically phenotypes of ClC-2 KO mice—were described in patients with ClC-2 mutations.

Crystal structures of prokaryote and eukaryote ClC proteins have provided important insights about molecular structure and ion conductance mechanisms. ClC proteins are unique in their double-barreled structure, providing a new model of ion transport in which the same basic architecture supports bona fide channel conductance and ion co-transport. These two types of ion translocation were believed to occur by entirely distinct mechanisms. However, the available crystal structures were not sufficient to uncover the molecular mechanism governing the common gating mechanism and the precise proton transport pathway. Use of new approaches or the development of novel techniques may be necessary to uncover the molecular mechanisms underlying ClC ion transport.

Generation of crystal structures of mammalian ClC channels and exchangers will ultimately permit a more accurate investigation into the differences between these two structures, and also the identification of regions involved in interaction and modulation by other cellular components. Moreover, those structures will greatly assist in the development of new compounds able to modify specific types of ClC proteins, thus opening the field for pharmacological approaches aiming at generating therapeutic drugs. Such drugs would have the potential to reduce or even eliminate the undesired symptoms caused by ClC proteins loss-of-function, improving quality of life for many patients.

## Author Contributions

DP analyzed the literature, wrote the paper, and prepared the figures; RP analyzed the literature and reviewed the paper; VC analyzed the literature, reviewed the paper and supervised the work.

## Conflict of Interest Statement

The authors declare that the research was conducted in the absence of any commercial or financial relationships that could be construed as a potential conflict of interest.

## Abbreviations

9-AC: 9-antracene-carboxylic acid
ABC transporters: ATP Binding Cassette transporters
CBS: cystathionine-β-synthase
cmClC: Cyanidioschyzon merolae ClC protein
CPP: p-chloro-phenoxy-propionic acid
DPC: N-phenylanthranilic acid
ecClC: Escherichia coli ClC protein
NAD: nicotinamide adenine dinucleotide
NFA: niflumic acid
PTH: parathyroid hormone
NPPB: 5-nitro-2-(3-phenylpropylamino) benzoic acid
OADS: 4,4′-octanamidostilbene-2,2′-disulfonate
PKC: protein kinase C
PTCs: proximal tubule cells
stClC: Salmonella enterica serovar typhimurium ClC protein
VRAC: volume-regulated anion channel
